# Widespread rash in a 45-year-old woman after moxifloxacin administration

**DOI:** 10.1016/j.jdcr.2024.08.043

**Published:** 2024-09-24

**Authors:** Fortunato Cassalia, Enrico Cocchi, Stefano Palo, Carmine D’Acunto, Davide Melandri

**Affiliations:** a Dermatology Unit, Department of Medicine (DIMED), University of Padua, Padua, Italy; b Neonatal and Pediatric Intensive Care Unit, AUSL Romagna, Ravenna, Italy; c Dermatology Unit and Burn Center, AUSL Romagna, Cesena Hospital, Cesena, Italy; d Department of Medical and Surgical Sciences, AUSL Romagna, Alma Mater Studiorum - University of Bologna, Bologna, Italy

**Keywords:** anti-TNF-alpha, Etanercept, Lyell's syndrome, moxifloxacin, SJS, SJS/TEN, TEN, toxic epidermal necrolysis

## Case presentation

**Fig 1:**
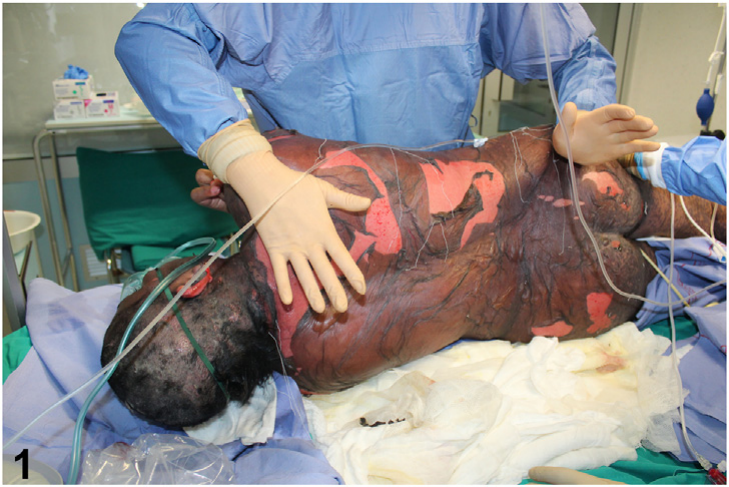

**Fig 2:**
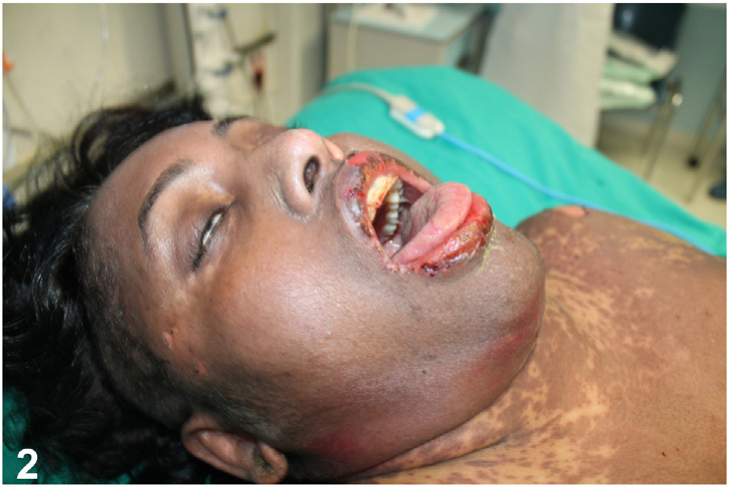

A 45-year-old African woman presented with fever, throat pain, and a rapidly deteriorating rash after taking moxifloxacin for 10 days. She exhibited extensive erythematous and purpuric macules, blisters, and significant mucosal involvement, covering 40% of her body surface area (Figs 1 and 2).

**Question 1: What is the most likely diagnosis?**

A. Toxic epidermal necrolysis (TEN)
B. Stevens-Johnson syndrome
C. Staphylococcal scalded skin syndrome
D. Erythema multiforme major
E. Pemphigus vulgaris

**Answers:**

A. TEN or Lyell's syndrome – Correct. This diagnosis is supported by the extensive epidermal necrosis involving more than 30% of the body surface and the significant mucosal involvement observed in the patient. TEN is a serious and life-threatening dermatological condition characterized by diffuse erythematous and purpuric maculae aggregating into large areas of skin detachment. The presence of Nikolsky's sign, where slight mechanical pressure causes further skin detachment, is indicative of the severe epidermal damage observed in TEN. Given these clinical features and the patient's history of moxifloxacin use, TEN is the most appropriate diagnosis.^1^^,^^2^
B. Stevens-Johnson syndrome – Incorrect. This involves less than 10 percent of the body surface.
C. Staphylococcal scalded skin syndrome – Incorrect. This typically spares mucosal surfaces and can be excluded based on the extensive involvement and mucosal lesions in this patient.
D. Erythema multiforme – Incorrect. Erythema multiforme major usually presents with less extensive target lesions and skin detachments.
E. Pemphigus vulgaris – Incorrect. This shows intraepidermal acantholysis on histopathology rather than the full-thickness epidermal necrosis seen in TEN.

**Question 2: What is the first step in the management of this patient’s condition?**

A. Discontinuation of moxifloxacin
B. Initiation of systemic corticosteroids
C. Administration of intravenous immunoglobulin
D. Application of topical antibiotics
E. Referral to a dermatologist

**Answers:**

A. Discontinuation of moxifloxacin – Correct. The first step in managing the patient's condition is to discontinue moxifloxacin. In cases of TEN, it is essential to discontinue the culprit drug immediately to prevent further progression of the condition. This step is crucial because continuation of the drug may exacerbate immune-mediated destruction of keratinocytes, resulting in increased morbidity and mortality.^3^
B. Initiation of systemic corticosteroids – Incorrect. Systemic corticosteroids may be considered, but they are not the initial step. The primary goal is to remove the trigger, which in this case is moxifloxacin, to stop the adverse reaction.
C. Administration of intravenous immunoglobulin – Incorrect. Also, intravenous immunoglobulins and other immunomodulatory treatments may be considered, but the primary goal is to stop moxifloxacin.
D. Application of topical antibiotics – Incorrect. The application of topical antibiotics is not relevant to the systemic nature of the TEN and does not address the underlying cause.
E. Referral to a dermatologist – Incorrect. Referral to a dermatologist is important for complete treatment but should follow immediate discontinuation of the drug.

**Question 3: Which biologic drug could be used in the therapy of this condition?**

A. Etanercept
B. Secukinumab
C. Ustekinumab
D. Tocilizumab
E. Anakinra

**Fig 3:**
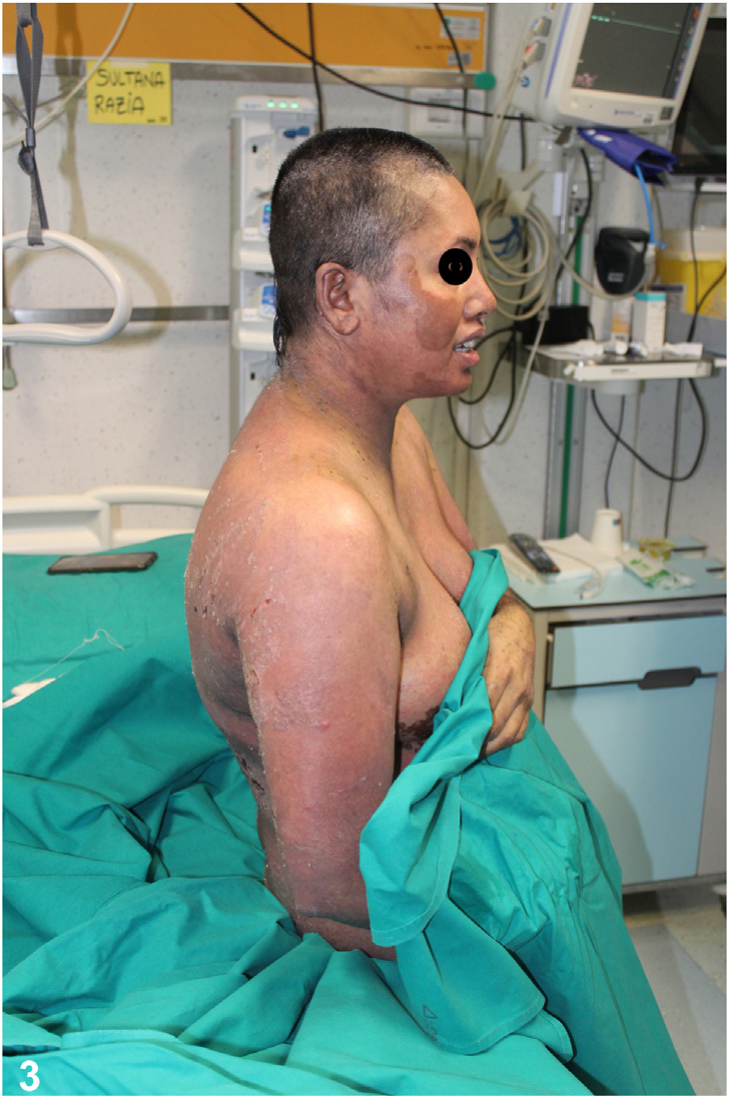
B. Secukinumab – Incorrect. Secukinumab targets interleukin (IL)-17 and is mainly used for diseases such as psoriasis and ankylosing spondylitis.
C. Ustekinumab – Incorrect. Ustekinumab targets both IL-12 and IL-23 and is effective for psoriasis and Crohn's disease.
D. Tocilizumab – Incorrect. Tocilizumab targets IL-6 and is used in conditions such as rheumatoid arthritis and cytokine release syndrome.
E. Anakinra – Incorrect. Anakinra targets IL-1 and is used for rheumatoid arthritis and certain autoinflammatory conditions.

**Answers**:

A. Etanercept – Correct. Etanercpet is a biological drug that could be used in the treatment of this disease. It is a tumor necrosis factor (TNF)-α inhibitor that can modulate the severe inflammatory response characteristic of TEN. By inhibiting TNF-α, a key cytokine involved in the pathogenesis of TEN, Etanercept may help reduce the extensive epidermal necrosis and inflammation. In this case, the patient in question received a single 50 mg dose of Etanercept subcutaneously, which led to significant clinical improvement within a week (Fig 3).^4^^,^^5^

## Conflicts of interest

None disclosed.
